# The Treatment of Croup

**Published:** 1870-11

**Authors:** 


The Treatment of Croup.---Prof. Fordyce Barker, at the request of Dr. Jacobi, has written out his views on this subject, which have been published in the *American Journal of Obstetrics and Diseases of Women and Children.* He thinks the terms false and true croup should be abandoned, as they convey a wrong impression; that the two diseases are identical, save in degree; that true croup is only a severer form of the same affection as "laryngismus stridulus," or "catarrhal laryngitis." But the croup of diphtheria he regards as an entirely distinct and specific disease.

In speaking of treatment, he urges an early resort to positive and active measures. He always commences the treatment with an emetic of the sub-sulphate of mercury (hydrargyri sulphas flava), three to five grains. If the first dose does not operate, he gives a second, but a third is hardly ever needed. This drug, for an emetic in croup; is superior to any other, on account of its promptness of action, because it is tasteless, and easily administered, and does not exhaust the system. The effect of an emetic is to deplete the mucous membrane by an abundant secretion of mucus, which is thrown off; to cause evacuation from the larynx, by the forced expiration, of any enudation, and to produce a revulsive effect.

Dr. B. urges the giving of this medicine very early in the disease; at once, upon the appearance of symptoms indicating any approach of croup. He advises that in families where there are young children with any tendency to laryngeal inflammation, there should be kept on hand powders of this emetic, to be used, in cases of emergency, by the parents or nurse, before the doctor's arrival. They can do no harm, if given occasionally when not really needed. If the case proves to be laryngitis stridulus, the source of the reflex-irritation should be sought and removed. If it is a case of simple catarrhal laryngitis, then opiates are the main reliance. But, to a case of true croup, when the child has a hot skin, quick pulse, and hurried breathing, Dr. B. prescribes veratrum viride, in doses of one to two drops of the tincture every two hours, visiting the child as often as every eighth hour, and regulating the dose, so that in a few hours the pulse is brought down to 80 per minute. He has used the veratrum for 25 years, and never found it fail to reduce the pulse of irritation or inflammation, and never saw any danger from its use when thus closely watched. If the indications are that the disease is extending downward, by thoracie rales, hurried and labored respiration, he adds to each dose of the veratrum two grains of carbonate of ammonia. Once in a while, when the laryngeal and trachial obstruction becomes great, he has repeated the emetic with the turpeth mineral on the second or third day. In the advanced stage of croup, when the respiration is hurried and irregular, the paroxysms of cough less marked, and the cough huskey, he finds great service from quinine, two or more grains every two hours. The tolerance and value of quinine in such cases is very great.

During the 20 years he has practiced in New York city, he has not lost a case of croup. This fortunate success is to be attributed to the promptness in treatment which he insists upon among the families he attends, in fact, that the remedy is always kept ready for immediate use; to the incessant care with which he watches the disease during its progress, and, in on small degree, to the special agents which he uses as remedies.

